# Scottish collaboration to integrate medicine therapy between out-patient and in-patient sectors

**DOI:** 10.1186/2052-3211-8-S1-K6

**Published:** 2015-10-05

**Authors:** Kenneth R Paterson

**Affiliations:** 1 University of Glasgow, Glasgow, G12 8QQ, UK

## 

Moves to harmonise good prescribing practice in Scotland (population 5.2 million) began two decades ago with the development of prescribing formularies in each of primary and secondary care in major geographically-based health boards. These formularies were then combined to produce ‘joint formularies’, the aim being to promote best and most efficient prescribing of evidence-based medicines in both the community and hospital settings.

Fifteen years ago, the challenges of new, and often expensive, medicines were addressed by creating a national health technology assessment organization (Scottish Medicines Consortium (SMC)) to undertake rapid assessment of the clinical effectiveness and cost-effectiveness of all new medicines. The SMC involves clinicians (doctors and pharmacists) from both primary and secondary care and from all across Scotland, ensuring short lines of communication to and from the prescribing ‘front line’. Clinical guideline development (by the Scottish Intercollegiate Guidelines Network (SIGN)) was already an active process, again involving clinicians from both sectors in all guidelines.

Ten years ago, with these systems in place and to avoid ‘mixed messages’ to the prescribing community, considerable efforts were made to develop ‘rules of engagement’ to ensure consistency across all prescribing advice. Only new medicines accepted for use by the SMC may be included in local formularies, and SIGN clinical guidelines will also only recommend use of SMC-approved medicines. This approach aims to maximize the consistency of prescribing advice and practice across the country.

Monitoring of adherence to advice has been undertaken locally and generally with a ‘light touch’, but ongoing developments are seeing the introduction of more detailed monitoring using centrally-held computerized prescribing data, particularly in primary care in the first instance.

The collaborative process has been evolutionary and took over 10 years to get from first steps to the present fully-integrated system. The process has been characterized by full involvement of clinicians throughout, with clinician leadership and ownership of the individual components such as formularies, the SMC and the SIGN. Other stakeholders, including the public, patient advocacy groups and the pharmaceutical industry are involved in parts of the process as appropriate.

Although established to encourage high-quality prescribing, the processes have also proved very useful in promoting cost-effective prescribing and in helping to achieve the best use of limited resources in both primary and secondary care. The expenditure required to develop and maintain the processes has been modest compared with the gains achieved and thus may represent a useful model for other small/medium-sized healthcare systems.

